# Thermoformability study of wood flour–HDPE composites with variations in wood content under vacuum forming

**DOI:** 10.1016/j.heliyon.2023.e22174

**Published:** 2023-11-10

**Authors:** Sanaz Afshariantorghabeh, Timo Kärki, Ville Leminen

**Affiliations:** Lappeenranta-Lahti University of Technology LUT, Mechanical Engineering, Yliopistonkatu 34, 53850, Lappeenranta, Finland

**Keywords:** Wood-plastic composite, Thermoforming, Vacuum forming, Thermoformability

## Abstract

Wood–plastic composites (WPCs) have emerged as sought-after substitutes for plastic. However, their use is restricted primarily to linear profiles owing to the lack of techniques for their three-dimensional (3D) forming. Thermoforming is a potential method for the 3D formation of WPCs. Accordingly, the aim of this study was to evaluate the thermoforming behaviour of extruded wood flour–high-density polyethylene composites with two different wood flour compositions (34 % and 54 %) under vacuum forming. This study examined the thermal behaviour of each structure during the forming process and the shape conformability, shape consistency, and surface quality of the formed samples. The findings suggest that increasing the quantity of plastic in composites can improve the obtained shape dimensions. Nevertheless, this improvement is accompanied by an increased level of inconsistency in the acquired profiles due to the distinct stretching rates of plastic and wood. Furthermore, the results reinforced the reliability of thermal analysis as a credible means of evaluating quality.

## Graphical abstract

**Figure:**
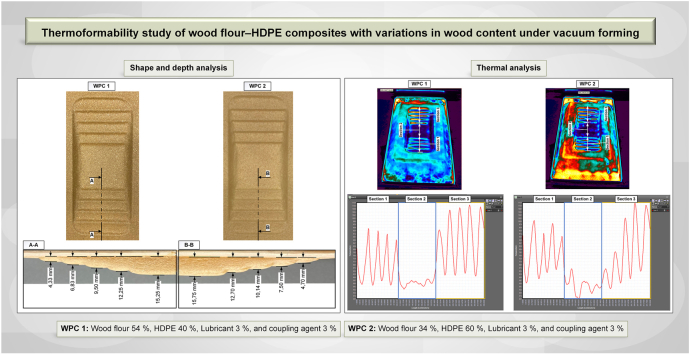

## 1 Introduction

Industrial applications are increasingly using composite materials fabricated from natural fibres and plastics. Wood–plastic composites (WPCs) are such types of composites created from hardwoods or softwoods, frequently discarded or as by-products, encapsulated in thermoplastic or thermoset matrixes [1]. WPCs have numerous advantages over conventional materials, including greater strength and durability, improved sustainability, and lower cost [[2], [3]]. The use of WPCs has traditionally been confined to the building and construction industry; however, it has expanded into other sectors such as automotive and packaging [4].

WPCs can be tailored to produce various properties by altering the ratio of wood fibres to plastic polymers and by incorporating additives to the mix. Because of the hydrophobic nature of plastics and the hydrophilic nature of woods, coupling agents are also used to produce good bonds between composites and enhance their different properties [5]. Studies have indicated that the wood content in WPCs can be reach up to 80 % [[6], [7]]. However, increasing the share of wood content in WPC may adversely affect the processability and end use of the resulting products owing to decreased moisture resistance [8].

Typically, a WPC is manufactured by extruding it into sheets or long profiles and then post-processing it to achieve more complex shapes [[9], [10], [11]]. Nevertheless, a challenge associated with WPCs is the limited number of methods available for post-forming them into versatile three-dimensional (3D) shapes compared with conventional materials such as plastics [12]. Injection moulding [[13], [14]], rotational moulding [15], and compression moulding [[16], [17]] are the most common processes used to produce 3D profiles of more versatile WPC shapes. A few studies have investigated the 3D forming of WPCs using press forming and hot-pressing techniques [[18], [19], [20], [21], [22]].

The restricted availability of 3D forming methods for WPCs can limit their applications and curtail design freedom. WPC production rates and market share must be further increased by introducing other manufacturing processes into this field. Thermoforming is a processing method that involves softening a material by heating and applying a vacuum, air pressure, or mechanical force to conform the material to the shape of the mould [23]. The thermoforming process has several advantages, including the possibility of high-volume production with reasonable tooling and production costs for a wide range of product sizes and shapes, which merit further investigation to determine the feasibility of producing different types of WPC products using this method [24].

A few studies have investigated the thermoforming of WPCs with different compositions. In a recent study, Friedrich [25] utilized a compolytics approach by combining polymer research and socio-technological investigations that were previously presented in WPC research. The aim was to construct a total effect model for thermoforming, and a decision tree consisting of a set of production parameters customized to suit the needs of specific target groups. Bhattacharyya et al. [26] thermoformed wood-fibre–polypropylene composites using various configurations, such as v-bending and matching die forming. Dobah et al. [27] investigated the influence of thermoforming process parameters on the thermoformability of flax reinforced polypropylene composites. Nevertheless, other studies have investigated the thermoforming of wood and polyethylene compositions, potentially owing to their ease of processing and lower degree of brittleness compared with polypropylene [28]. In particular, high-density polyethylene (HDPE) has attracted significant interest owing to its high rigidity, with research primarily relying on numerical simulations to confirm the feasibility of thermoforming HDPE–wood composites [[29], [30], [31]].

Of the studies mentioned, two focused on the thermoforming of WPC structures by applying a mechanical force ⦋ [25,27]⦌, whereas the other three examined thermoforming combined with free air blowing before the actual process [[29], [30], [31]]. Nevertheless, simple vacuum forming, which is cost-effective and widely available, has been relatively understudied in this field. Therefore, further experimental research should be conducted on this composition to gain a broader understanding of the possibility of vacuum thermoforming WPCs that may have various industrial applications.

Therefore, the aim of this study was to experimentally investigate the thermoforming behaviour and thermoformability of extruded wood flour–HDPE composites under vacuum forming. This study analysed the thermal behaviour in the forming process to examine the thermoforming behaviour and evaluate the shape conformability, shape consistency, and surface quality of the formed samples to investigate their thermoformability. Two distinct wood flour proportions (34 % and 54 %) were selected to demonstrate the differences in thermoformability based on the proportion of wood in composition. The selection of the wood content range for this study was informed by the typical wood content found in practical wood plastic industry applications, which typically falls between 40 wt% and 60 wt% in polyolefins [32]. In this research, the lower and upper limits of abovementioned range were selected, while also considering the inclusion of 6 % additives, based on empirical experience. Furthermore, this study particularly focused on thin-gauge composites with a thickness of 2 mm to determine the feasibility of manufacturing thinner products from WPCs with sufficient structural integrity and consistent quality. The objective was to create a foundation for various applications beyond those in the construction and automotive industries.

## 2 Experimental

### 2.1 Material preparation

This study examined wood flour–HDPE composite sheets containing two different compositions of wood and plastic (Table 1). Spruce wood flour was prepared at the Fiber Composite Laboratory (LUT University, Finland) using a crushing machine and hammer mill and sieved with a 20 mesh. Hostalen GC7260 from LyondellBasell was used as the HDPE with a melt mass-flow rate of 23 g/10 min. The structures also included the Struktol TPW 113 lubricant, and the coupling agent used was malleated polypropylene Fusabond E226 from DuPont.

**Table 1:** Composition of used composites for the study.

Material	Wood flour (%)	HDPE (%)	Lubricant (%)	Coupling agent (%)
WPC 1	54	40	3	3
WPC 2	34	60	3	3

The materials were combined using a PLASMEC TRL100/FV/W turbomixer and a PLASMEC RFV-200 cooler before being extruded into sheets using a Weber CE7.2 twin-screw extruder. WPC 1 was extruded at 189 °C with a screw speed of 23 rpm, and WPC 2 was extruded at 162 °C with a screw speed of 30 rpm. The pressure at the die ranged from 60 to 147 MPa, and the material output varied between 8 and 9.5 kg/h depending on the materials used. Composites were prepared with random orientation of fibers within the matrix aiming to achieve isotropic properties. The resulting WPC sheets were sanded to an average thickness of 2 mm for thermoforming.

### 2.2 Thermoforming process

**Fig. 1:**
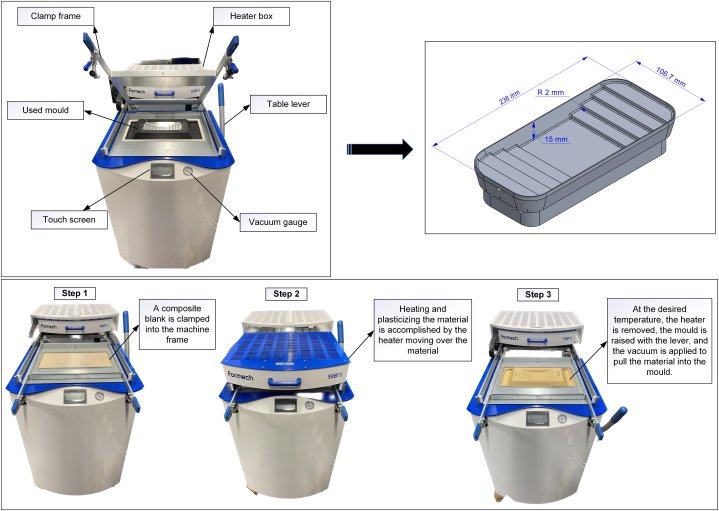
Demonstration of the used thermoforming machine, mould, and forming steps.

In this study, composite sheets were 3D formed using a vacuum-forming process. Vacuum forming is a type of thermoforming in which a heated and softened material is shaped into a mould by applying a vacuum between the mould and sheet [33]. This study used a floor-standing vacuum-forming machine (Formech 508DT/FS, Hertfordshire, UK) and an aluminium mould with varying depths and small radii to provide better insight into the shapability of each tested composition. The mould was placed into the machine and secured with a lever on the right side. As shown in Fig. 1, the mould was placed in a plastic block to prevent the material from being pulled beneath the mould during the forming process.

The initial stage of forming involved placing a composite blank (260 mm × 460 mm) in a clamp frame (step 1 in Fig. 1). When the heater moved over the material, it was heated and plasticised (step 2 in Fig. 1). Once the material reached the required temperature, the heater was removed, the lever was raised, and a final vacuum was applied to pull the material into the mould, where it was cooled and hardened, preserving its shape (step 3 in Fig. 1). Note that there was a short delay between removing the heater and mould coming into contact with the heated sheet, which was subject to the performance of the operator.

The machine used was designed for prototype construction, which caused unavoidable variations. The heating time before vacuuming is one such variation, in which the available functions of the machine do not support the selection of the heating time required to reach a specific temperature. Instead, the material was heated based on the specific heater capacity, which was set to 75 % during this investigation. Accordingly, the heating time in each cycle needed to achieve the required temperature depended on the cycle number and the duration of heater deactivation. Therefore, it was necessary to monitor the temperature during heating to determine when it reached the desired level. The temperatures of the sheets were measured using a thermal camera (FLIR A8201sc, Täby, Sweden).

To compare the thermoformability of two composite compositions, we subjected them to the same forming temperature of 160 °C. Preliminary analysis determined this temperature to be optimal because temperatures below 160 °C resulted in unsatisfactory forming outcomes for both compositions, whereas temperatures above this value led to material degradation. Because of the inhomogeneous nature of the material, it was impossible to attain the same temperature across the entire blank. Therefore, an average temperature of 160 ± 5 °C was selected for the blank area. Additionally, the process utilized a fixed vacuuming pressure (6 in HG) to ensure consistency throughout the testing.

### 2.3 Material characterization methods

The thickness and tensile and impact properties were measured before the experiments on the specimens, which were maintained at 23 ± 1 °C and 50 % relative humidity for one week. The thickness of the specimens was measured using a handheld digital calliper to ensure the required thickness of 2 mm after extrusion and sanding. Tensile and impact properties were measured due to their potential effect on forming performance and the damage mechanisms that may arise during the thermoforming process. It is of utmost importance to emphasize that the purpose of conducting measurements was to characterize the produced materials and acquire a more profound comprehension of the observations made throughout the forming process, rather than predicting their performance during processing.

Tensile testing was performed according to ISO 527 using a Zwick/Roell Z020 tester with an extensometer (Germany). Samples were prepared in a dog-bone shape, and testing was performed on 12 parallel samples for each composition using a 10 N preload and 1 mm/min test speed. With a Zwick 5102 model impact tester (Germany) and the ISO 179-1/1f U standard, the Charpy impact strength of the unnotched samples was determined in a flatwise position for 20 sample replicates for each composition. This method is based on the recommendations in TS 15534-1 “Wood-plastic composites (WPC)-Part 1: Test methods for characterizing WPC products and materials.” A pendulum impact tester with a 5 Kpcm hammer size was used, and the Charpy impact strength was computed using Equation (1), where Ec is the energy absorbed by the test specimen (joules), h and b are the thickness of the test specimen (mm) and the width of the test specimen (mm), respectively.

1 $$\text{acU}=\frac{\text{Ec}}{h\times b}\times 103$$

### 2.4 Thermoforming analysis methods

In this study, thermal, shape conformability, and surface roughness analyses were used for comparative analysis of the thermoforming process of the respective materials and thermoformability differences between the two studied structures. Ten samples were produced for each structure to ensure reliability and repeatability of the obtained results. *Thermal analysis* was performed using a thermal camera (FLIR A8201sc, Täby, Sweden) with a resolution of 1024 × 1024 and a sensitivity below 20 mK. Using a 25 mm lens, the study was conducted in the temperature ranges of 10–90, 30–150, and 150–350 °C. The entire forming process, from fixing the material into the clamps to the completion of forming and cooling down of the formed parts, was recorded. FLIR Research IR analysis software was used to analyse the obtained temperature distributions.

The *shape conformability* and *surface roughness* of the samples were analysed using a 3D measurement system (Keyence VR-3200, Osaka, Japan). The system obtained 3D shape profiles and measured the required dimensions of the shapes using an analysis software. The surface roughness was measured using the same system with a 50 × low magnification camera using multiline roughness with 30 lines and 4-pixel intervals to measure Rz, which is the difference between the tallest peak and deepest valley on the surface. A handheld digital calliper was used for *depth measurements*.

## 3 Results and discussions

### 3.1 Materials properties

Generally, strength properties of wood plastic composites can be influenced by several factors including length and type of fibre material, fibre content, type of polymer matrix, interfacial adhesion between fibre and matrix, and their processing method [[34], [35], [36]]. This study specifically concentrated on the tensile and impact properties, considering their potential impact on the thermoforming behavior of materials. Notably, the dependence of the thermoforming process on tensile properties has been underscored in various studies involving diverse materials. For instance, in plastic-coated fibre-based composites, research has demonstrated a direct correlation between tensile strength and the depth achieved during the process, while elongation plays a crucial role in determining the final shape [24]. Though coated paperboards differ from wood-plastic composites (WPC) in structure, their wood-based nature remains relevant for consideration. Additionally, another research highlighted the importance of impact strength in relation to breakage and fracture propagation in fiber-reinforced composites, with an investigation into the influence of fiber content on this property [37]. Given the possibility of localized stresses in the thermoforming process, which could serve as potential spots for crack propagation, the relevance of impact properties is particularly notable in such scenarios.

Table 2 presents the measured properties of the fabricated WPCs. WPC 2 had an impact strength 1.5 times higher than WPC 1, which was consistent with earlier studies indicating that increasing the fibre content reduces the Charpy impact strength of WPCs [38]. This is due to the reduced toughness resulting from the inclusion of more wood content, limiting polymer chain mobility, and leading to lower energy absorption capacity [39]. Additionally, increasing the wood content weakens the interfacial adhesion, creating more voids for crack propagation and reducing the impact strength ⦋40⦌. The tensile test results also indicated higher strength and elongation values for WPC 2 than those for WPC 1. This was likely due to the weaker interfacial bonding between the hydrophilic wood and hydrophobic plastic structures at higher wood content ⦋ [40,41]⦌. Furthermore, the lower elongation at break with increasing fibre content can be attributed to the inherently lower elongation properties of wood compared with plastic materials [42].

**Table 2:** Mechanical properties of studied composites.

Material	Impact strength (kJ/m^2^)	Tensile strength (MPa)	Modulus of elasticity (GPa)	Elongation at break (%)
WPC 1	2.69 *±* 0.43	9.49 *±* 2.20	1.79 *±* 0.33	0.40 *±* 0.10
WPC 2	4.16 *±* 0.39	15.26 *±* 0.99	2.15 *±* 0.17	0.90 *±* 0.10

### 3.2 Thermal analysis of the forming process

**Fig. 2:**
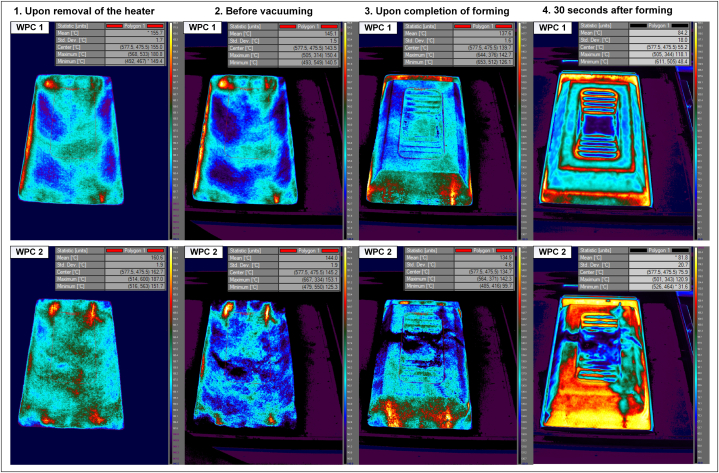
Temperature distribution of WPCs 1 and 2 throughout the forming procedure.

A thermal camera was used to record the forming process at different stages to gain a better understanding of the procedure, its limitations, and the forming results by analysing the temperature distributions. Before the forming process began, both materials were checked to ensure that they had a room temperature of 26 °C throughout. The materials were then heated to reach the desired temperature of 160 °C. Fig. 2 shows the subsequent stages of the process after the heater was removed until completion of the forming process, and the material was left to cool for 30 s.

A wide range of temperatures was involved throughout the process, which necessitated the use of different temperature ranges for the thermal recording. This resulted in the colours displayed in each stage not representing the same temperature in the next stage; this was also indicated by the temperature bars. However, the temperature bars in each step were the same for both materials. The polygon area shown in Fig. 2 corresponds to the mould region in the images, which was the area of interest.

The design of the forming device resulted in a time gap between the removal of the heater and the raising of the mould for forming. Accordingly, the temperature distribution was measured directly after the removal of the heater (step 1) and immediately before vacuuming (step 2). The time interval in this study was an average of 5 s, which was dependent on the operator performance. As shown in Fig. 2, this time gap caused a loss of heat and a decrease in temperature in both materials because there was no insulation, and the materials were at room temperature. Therefore, it follows that forming does not occur at the target temperature but at a lower temperature. A previous study observed similar heat loss when studying the thermal behaviour of continuous fibre-reinforced thermoplastic composites during thermoforming [43]. Their findings indicated that the heater capacity affects the rate of temperature loss, where high heating power leads to shorter heating durations and faster cycle times, whereas low heating power enables more time for the heat to soak into the depth of sheets, resulting in a slower rate of heat loss [43].

As shown in Fig. 2, both WPC 1 and WPC 2 experienced a decrease in temperature during the thermoforming process, with WPC 2 experiencing a larger temperature decrease than WPC 1. The average temperature drops were 10 °C for WPC 1 and 16 °C for WPC 2. Furthermore, WPC 2 exhibited greater temperature changes during most of the process, which could be due to its higher plastic content and greater thermal conductivity [1].

As shown in Fig. 2, the temperature distribution in the blank sheet was not uniform. As confirmed in preliminary testing with homogeneous plastic, part of the unevenness was caused by the nonuniform heating of the material. Nevertheless, other factors could have contributed to this problem, which may be attributed to the nonuniformity resulting from the heterogeneous composition of the composites, as also observed in previous research on composite structures [44]. Both compositions exhibited nonuniform temperature distributions, but WPC 2 exhibited higher nonuniformities after forming, particularly in step 4. Before forming, the standard deviations (STDs) of the average values for both compositions were similar. However, in steps 3 and 4, the STD of WPC 2 increased. Note that despite the small variations that may be attributed to the natural material properties, the same results were observed in all repetitive samples.

**Fig. 3:**
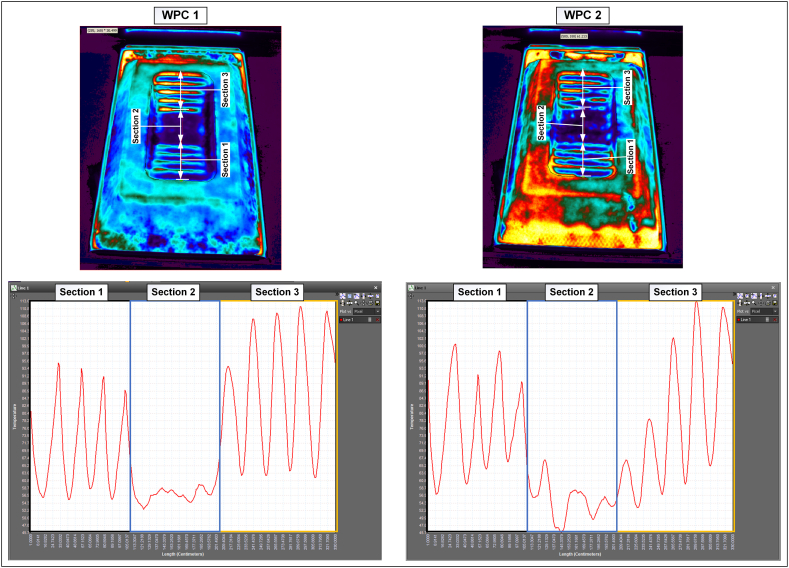
Temperature profiles over the midplane length line.

To demonstrate the validity of the observed results, the temperature profile of the midline of the length in step 4 was analysed for both compositions, and the results are presented in Fig. 3. The samples were divided into three areas to clarify the temperature differences. The hill-shaped profiles of areas 1 and 3 corresponded to each step of the stair-shaped mould. A series of steps have the same length and radius but occur at varying depths. Accordingly, depending on when the material reaches a particular depth, the peak temperature of each hill-shaped profile may vary; however, the hill shape should ideally remain the same during complete forming. The hill shapes in WPC 1 were more similar to each other than those in WPC 2, where more variations were evident. Furthermore, in area 2 of the sample, which was the flat part at the bottom, a horizontal line should have been observed under ideal conditions. However, owing to the nonuniformity of the structure, this line was not flat for both compositions, and WPC 2 exhibited more variations and nonuniformity than WPC 1.

The greater nonuniformity with a higher plastic content in the composites can be ascribed to two factors: the difference in stretching rates between plastic and wood, and the distribution of wood fibers within the matrix. In composites featuring a lower proportion of wood, the even distribution of wood fibers across all regions within the matrix might not be achieved, potentially leading to heightened irregularity. Moreover, in composites with higher plastic content, plastic becomes the dominant phase during forming. This is followed by plastic greater capacity for stretching compared to the less stretchable wood fibers, leading to areas without reinforcement fibres in the final structure. Conversely, in composites containing a higher proportion of wood, fibers regulate the behaviour, resulting in fewer areas without fibres within the final structure and thus achieving greater consistency in the structure. In this study, the increased level of nonuniformity for WPC 2 can be primarily attributed to the different stretching rates of plastic and wood. This can be distinguished by observing the close standard deviation values shown by WPC 1 and 2 in Fig. 2 before thermoforming, suggesting little variation between composites and consequently, an acceptable level of distribution in WPC 2.

### 3.3 Shape conformability analysis

**Fig. 4:**
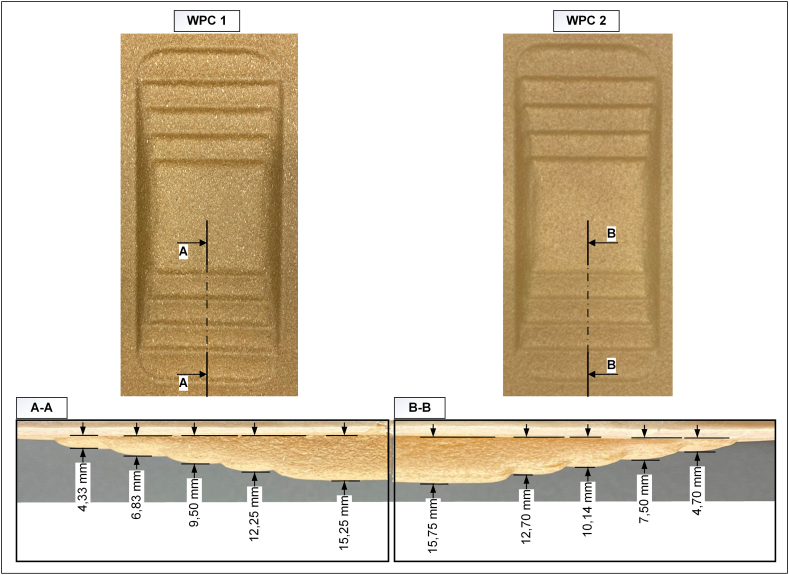
Visual presentation of the obtained samples from each studied composition.

This section investigates the shape conformance of materials as an indicator of their thermoformability. The samples were visually inspected, and their depths were subsequently measured. A 3D analysis was then conducted to compare the measured values of the specimens with those of the mould. Both samples achieved the designed depths of the mould in each step, which were 3, 6, 9, 12, and 15 mm (Fig. 4). The slight variation in the values obtained could be related to both the measurement error and nonuniformity of the surface flatness. In a previous investigation, it was observed that spring-back occurred during the thermoforming process of multilayer fiber-based composites [45]. However, the attainment of an identical depth as the mould for these mixed structure WPCs could indicate the absence of significant spring-back for this particular geometry and the employed process.

**Fig. 5:**
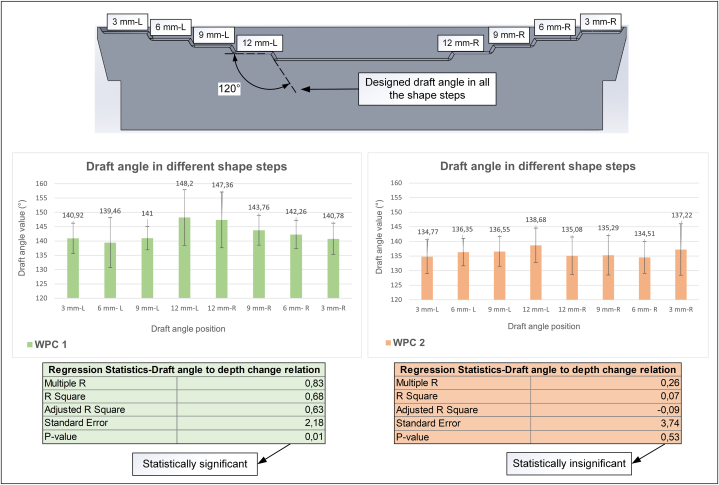
Shape conformability analysis of the studied materials.

For a more accurate understanding of the extent of formability, the 3D shapes of the samples were obtained, and the corresponding draft angles in each step (originally 120° in the mould design) were measured. The draft angle was selected as the shape-formability factor because when the material is more formable, it can be drawn more thoroughly into a shape, enabling the angles of the shape to be more accurately replicated. The draft angle was measured at each depth step of the shape because deeper depths may make it more difficult for the material to conform to. Fig. 5 shows the average values of five parallel samples obtained for each composition.

Fig. 5 demonstrates that WPC 2 could achieve lower angles than WPC 1, with a difference of approximately 3°–10°, despite the change in depth. These findings corresponded with the 2D tensile properties presented in Table 1, where WPC 2 exhibited a higher strain at break, which could potentially correspond to better stretching and shape replication ability. However, the symmetry of the structure was noteworthy. In WPC 1, a strong linear relationship existed between the obtained angle and depth change throughout the entire structure, regardless of whether it was on the left or right side of the tray. In contrast, WPC 2 lacked such a correlation and less uniformity was evident in the achieved angles. These findings reinforced the thermal analysis results, confirming the low consistency and uniformity of the structure of WPC 2.

The overall analysis of shape conforming exhibited satisfactory forming rates for both composites in the given geometry. Nevertheless, increasing the plastic content can enhance the shape conformance in such a forming process, where material stretching is the primary factor influencing deformation while with compromising uniformity. A previous study on thermoforming HDPE-based composites using air blow forming reported that successful thermoforming can be achieved with a wood content of 40 % or less as an increase in wood content results in a more elastic composite in forming [31]. Nevertheless, the results of this study revealed a promising forming rate with 54 % wood content in the structure without any crack occurrence.

It is also worth noting that increasing the fiber content within composite materials may potentially lead to an elevation in the level of porosity and the volume of pores within the structure [46]. This, in turn, can influence on various factors in thermoforming process, including the vacuum application, heat distribution, and the occurrence of cracks. Essentially, the presence of pores within the material can function as air channels, serving as venting holes within the moulds, which may optimize vacuum process implication and impacting the forming rate. Furthermore, pores could potentially serve as vulnerable points for crack propagation. Moreover, the level of porosity and the presence of additional air within the structure can also influence the distribution of heat throughout the material and subsequent temperature variations.

However, in this research, when evaluating the forming rate in these two structural variations, both exhibited satisfactory forming rates without any instances of cracking. Moreover, considering the temperature distributions, as illustrated in Fig. 2, an equivalent level of average temperature and deviations can be observed for both structures prior to the forming process. Consequently, within the parameters of this study, the level of porosity may not bear a significant effect on the selected level of forming. One explanation for this observation is that the change in fiber content rate is not substantial enough to yield a significant variance in porosity. It is worth considering that porosity might wield a more pronounced influence in a more demanding structural and forming context.

### 3.4 Surface roughness

It is crucial to examine the surface roughness and uniformity across a product because they determine the viability of the process and the resulting product for specific applications [47]. Accordingly, the surface roughness of the non-sanded sheets was initially evaluated after extrusion and before thermoforming. Subsequently, it was measured after the thermoforming process in the direction of the length (parallel to the fibre direction) at various depths of the shape for five identical samples of each composition. Further, the 95 % confidence interval for surface roughness, derived from the mean and standard deviation of the data, were presented. It provides a level of confidence that the true population mean of surface roughness lies within this interval.

**Fig. 6:**
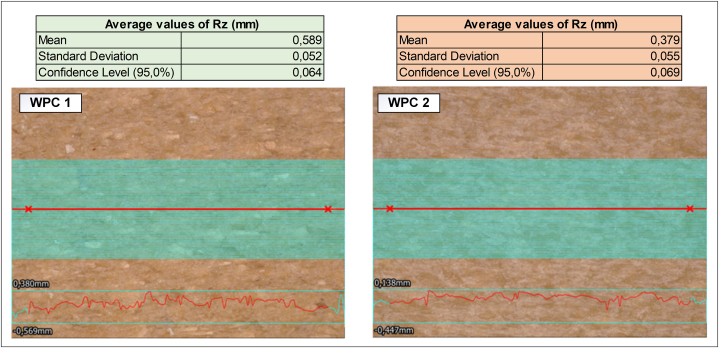
Surface roughness analysis of sheets before thermoforming.

The findings showed that prior to thermoforming, WPC 1, which had a higher proportion of wood, had a greater surface roughness than WPC 2 (Fig. 6). This outcome corresponded with a previous investigation of the WPC surface roughness with varying wood content. It was observed that when more plastic was used, the surface roughness decreased, likely due to the lower presence of internal void space in plastics and better surface quality compared with wood [48]. Both structures also exhibited variations in roughness as their thicknesses had not yet been uniformly sanded [49]. However, Fig. 6 illustrates that the variation level was nearly identical in both composites. This observation suggests a comparable level of uniformity in the structures before undergoing thermoforming. This is of particular importance, as elevated variations linked to higher wood content could potentially signal uneven dispersion or agglomeration.

**Fig. 7:**
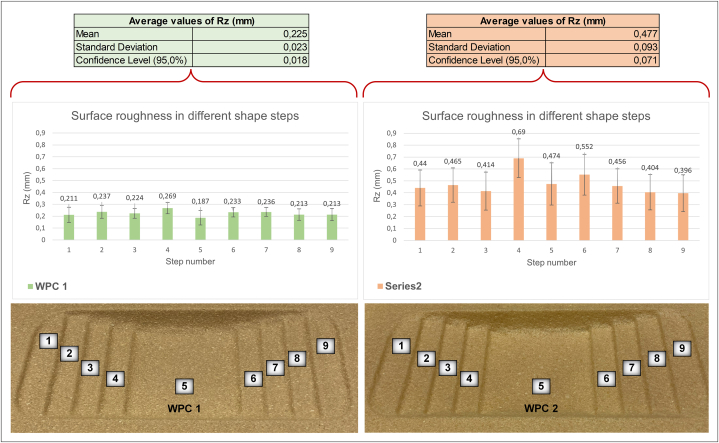
Surface roughness analysis in formed samples of each studied composition.

As shown in Fig. 7, the surface roughness was measured at different depths of the shape after thermoforming; however, because no statistically significant change was observed, an average value was used for comparison. The samples were sanded and smoothed before thermoforming, resulting in reduced roughness. Correspondingly, the results showed that the surface roughness and confidence level decreased for WPC 1 after forming, but surprisingly, it increased for WPC 2. Additionally, the confidence level was higher for WPC 2 than for WPC 1. Despite the close values of confidence level for both materials before forming, the difference range increased after forming. The reason for this behaviour could again be the differences in the stretching behaviour of the fibres and plastic, resulting in different thicknesses and variations in the surface structure.

**Fig. 8:**
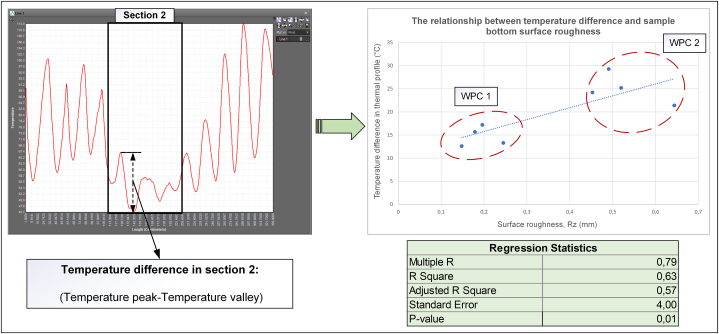
Statistical comparison between temperature difference and roughness in the bottom flat area of samples.

The nonuniformity in the thermal analysis was confirmed using a separate analysis of the shape conformability and surface roughness. Interestingly, another statistical analysis conducted on the findings demonstrated a positive correlation between the thermal profiles and the surface roughness of the samples. Considering section 2 in Fig. 8, a strong relationship occurred between the difference in the peak and valley temperatures of the thermal profile after forming and the Rz roughness of the same area, which was again the difference between the peak and valley roughness. As shown in Fig. 8, the thermal changes for WPC 1 exhibited less differences, which correlated with the lower roughness compared with WPC 2, implying higher values for both variables. This result confirmed the validity of thermal analysis as a productive indicator of quality.

## 4 Conclusions

In this study, the thermoforming behaviour of thin-gauge extruded wood flour–HDPE composites with two different wood flour proportions (34 % and 54 %) was investigated under vacuum forming. The thermoformability was assessed using thermal analysis, shape conformability, shape consistency, and surface roughness.

The findings revealed that both composites exhibited satisfactory forming rates with the same forming configuration, including the starting thickness, forming temperature, heating power capacity, and vacuum application in the given geometry. Nevertheless, increasing the plastic content can enhance the shape conformance in such a forming process, where material stretching is the primary factor influencing deformation. Conversely, although increasing the plastic content in composite structures can lead to better shape conformance, it is important to recognize that wood fibres might not achieve uniform distribution throughout all regions. In addition, wood fibers are further limited in their ability to stretch along with the plastic. Consequently, a higher degree of nonuniformity is exhibited in such a structure, as evidenced by the inconsistent temperature distribution, shape symmetry, and surface roughness throughout the geometry.

Furthermore, the findings demonstrate the credibility of thermal analysis as a reliable method for evaluating the quality of thermoforming processes. The focus of this study was primarily to evaluate thermoformability from a forming perspective, and future research can expand upon this by analysing the functional performance of the resulting structures.

## Data availability

Data will be made available on request.

## CRediT authorship contribution statement

**Sanaz Afshariantorghabeh:** Conceptualization, Formal analysis, Investigation, Methodology, Validation, Visualization, Writing – original draft, Writing – review & editing. **Timo Kärki:** Conceptualization, Formal analysis, Methodology, Supervision, Validation, Writing – review & editing. **Ville Leminen:** Conceptualization, Formal analysis, Methodology, Supervision, Validation, Writing – review & editing.

## Declaration of competing interest

The authors declare that they have no known competing financial interests or personal relationships that could have appeared to influence the work reported in this paper.

## References

[1] Teaca C.A., Tanasa F., Zanoaga M. (2018). Multi-component polymer systems comprising wood as bio-based component and thermoplastic polymer matrices-an overview. Bioresources.

[2] Thompson D.W., Hansen E.N., Knowles C., Muszynski L. (2010). Opportunities for wood plastic composite products in the US highway construction sector. Bioresources.

[3] Malkapuram R., Kumar V., Negi Y.S. (2009). Recent development in natural fiber reinforced polypropylene composites. J. Reinforc. Plast. Compos..

[4] Partanen A., Carus M. (2016). Wood and natural fiber composites current trend in consumer goods and auto- motive parts. Reinforc Plast.

[5] Ndiaye D., Verney V., Askanian H., Commereuc S., Tidjani A. (2013). Morphology, thermal behavior and Dy- namic rheological properties of wood polypropylene composites. Mater. Sci. Appl..

[6] Roig I. (2018). Biocomposites for interior facades and partitions to improve air quality in new buildings and restorations. Reinforc Plast.

[7] Gacitua W., Bahr D., Wolcott M. (2010). Damage of the cell wall during extrusion and injection molding of wood plastic composites. Compos. Appl. Sci. Manuf..

[8] Wang Q., Ou R., Shen X., Xie Y. (2011). Plasticizing cell walls as a strategy to produce wood-plastic composites with high wood content by extrusion processes. Bioresources.

[9] Gonçalves N.D., Teixeira P., Ferrás L.L., Afonso A.M., Nóbrega J.M., Carneiro O.S. (2015). Design and optimization of an extrusion die for the production of wood-plastic composite profiles. Polym. Eng. Sci..

[10] Xiong W., Wu Q., Cai C.S. (2013). Mechanical and thermal performance of coextruded wood plastic composites for structural applications. Adv. Struct. Eng..

[11] Turku I., Hämäläinen K., Kärki T. (2014). Co-Extrusion of wood flour/PP composites with PP-Based cap layer reinforced with Macro-and Micro-Sized Cellulosic Fibres. Adv. Mater. Res..

[12] Toghyani A.E., Amraei M., Matthews S., Varis J., Kärki T., Zhao X.L. (2017). Effect of strain rate and temperature on press forming of extruded WPC profiles. Compos. Struct..

[13] Montanes N., Quiles-Carrillo L., Ferrandiz S., Fenollar O., Boronat T. (2019). Effects of lignocellulosic fillers from waste thyme on melt flow behavior and processability of wood plastic composites (WPC) with biobased poly (ethylene) by injection molding. J. Polym. Environ..

[14] Sohn J.S., Cha S.W. (2018). Effect of chemical modification on mechanical properties of wood-plastic composite injection-molded parts. Polymers.

[15] Martins C.I., Gil V., Rocha S. (2022). Thermal, mechanical, morphological and aesthetical properties of rota- tional molding PE/pine wood sawdust composites. Polymers.

[16] Sormunen P., Kärki T. (2019). Compression molded thermoplastic composites entirely made of recycled mate- rials. Sustainability.

[17] Chauhan V., Kärki T., Varis J. (2021). Design of tooling system and identifying crucial processing parameters for NFPC manufacturing in automotive applications. J. Compos. Sci..

[18] Matthews S., Toghyani A.E., Kłodowski A., Eskelinen H., Kärki T., Varis J. (2015).

[19] Matthews S., Tanninen P., Toghyani A., Leminen V., Varis J. (2022). Numerical evaluation of press forming parameters and mould geometry in wood plastic composite (WPC) products. Key Eng. Mater..

[20] Matthews S., Toghyani A., Tanninen P., Hyvärinen M., Leminen V., Kärki T. (2019). Effect of press force in tensile strength and surface quality of press formed wood plastic composite products. AIP Conf. Proc..

[21] Friedrich D. (2022). Post-process hot-pressing of wood-polymer composites: effects on physical properties. J. Build. Eng..

[22] Friedrich D. (2021). Thermoplastic moulding of Wood-Polymer Composites (WPC): a review on physical and mechanical behaviour under hot-pressing technique. Compos. Struct..

[23] Toghyani A., Matthews S., Varis J. (2019). Cutting repeatability of an extruded wood plastic composite in a post-production process. Proc. Manuf..

[24] Afshariantorghabeh S., Kärki T., Leminen V. (2022). Three-dimensional forming of plastic-coated fibre-based materials using a thermoforming process. Packag. Technol. Sci..

[25] Friedrich D. (2023). Thermoforming of wood-plastic composites: a compolytics-approach translating combined polymer and policy analyses into industrial design principles. Int. J. Adv. Des. Manuf. Technol..

[26] Bhattacharyya D., Bowis M., Jayaraman K. (2003). Thermoforming woodfibre-polypropylene composite sheets. Compos. Sci. Technol..

[27] Dobah Y., Zampetakis I., Ward C., Scarpa F. (2020). Thermoformability characterisation of Flax reinforced polypropylene composite materials. Compos. B Eng..

[28] Khanam P.N., Almaadeed M.A.A. (2015). Processing and characterization of polyethylene-based composites. Adv. Manuf. Polym. Compos. Sci..

[29] Erchiqui F., Godard F., Gakwaya A., Koubaa A., Vincent M., Kaddami H. (2009). Engineering investigations on the potentiality of the thermoformability of HDPE charged by wood flours in the thermoforming part. Polym. Eng. Sci..

[30] Erchiqui F., Godard F., Koubba A., Vincent M., Kaddami H. (2009). Investigation of relaxation properties and potentiality of the thermoformability of HDPE charged by wood flours. J. Reinforc. Plast. Compos..

[31] Sukiman M.S., Erchiqui F., Kanit T., Imad A. (2020). Design and numerical modeling of the thermoforming process of a WPC based formwork structure. Mater. Today Commun..

[32] Butylina S., Martikka O., Kärki T. (2010). Properties of wood fibre-polypropylene composites: effect of wood fibre source. Appl. Compos. Mater..

[33] Mansoor I., Naseer A., Qadeer A. (2022). Manufacturing of economical packing by using vacuum forming technique. Eng. Proc..

[34] Neher B., Bhuiyan M.M.R., Kabir H., Qadir M.R., Gafur M.A., Ahmed F. (2014). Study of mechanical and physical properties of palm fiber reinforced acrylonitrile butadiene styrene composite. Mater. Sci. Appl..

[35] Velmurugan R., Manikandan V. (2007). Mechanical properties of palmyra/glass fiber hybrid composites. Compos. Appl. Sci. Manuf..

[36] Migneault S., Koubaa A., Erchiqui F., Chaala A., Englund K., Wolcott M.P. (2009). Effects of processing method and fiber size on the structure and properties of wood-plastic composites. Compos. Appl. Sci. Manuf..

[37] Hernández-Díaz D., Villar-Ribera R., Espinach F.X., Julián F., Hernández-Abad V., Delgado-Aguilar M. (2020). Impact properties and water uptake behavior of old newspaper recycled fibers-reinforced polypropylene composites. Materials.

[38] Chauhan V., Kärki T., Varis J. (2021). Effect of fiber content and silane treatment on the mechanical properties of recycled acrylonitrile-butadiene-styrene fiber composites. Chemistry.

[39] Threepopnatkul P., Teppinta W., Sombatsompop N. (2011). Effect of co-monomer ratio in ABS and wood content on processing and properties in wood/ABS composites. Fibers Polym..

[40] Kim T.W., Lee S.Y., Chun S.J., Doh G.H., Paik K.H. (2011). Effect of silane coupling on the fundamental properties of wood flour reinforced polypropylene composites. J. Compos. Mater..

[41] Chotirat L., Chaochanchaikul K., Sombatsompop N. (2007). On adhesion mechanisms and interfacial strength in acrylonitrile-butadiene-styrene/wood sawdust composites. Int. J. Adhesion Adhes..

[42] Gu R., Kokta B.V., Michalkova D., Dimzoski B., Fortelny I., Slouf M. (2010). Characteristics of wood- plastic composites reinforced with organo-nanoclays. J. Reinforc. Plast. Compos..

[43] Wen Y., Xinying C., Ngoc D.L.Q., Bin T.L., Sze T.W. (2022).

[44] Bourai K., Riedl B., Rodrigue D. (2013). Effect of temperature on the thermal conductivity of wood-plastic composites. Polym. Polym. Compos..

[45] Afshariantorghabeh S., Pesonen A., Kärki T., Leminen V. (2023). Effects of thermoforming operation and tooling on the thermoformability of plastic‐coated fibre‐based materials. Packag. Technol. Sci..

[46] Pupure L., Varna J., Joffe R., Berthold F., Miettinen A. (2020). Mechanical properties of natural fiber composites produced using dynamic sheet former. Wood Mater. Sci. Eng..

[47] Toghyani A., Matthews S., Varis J. (2019). Effect of dwell time and press speed on the forming quality of the press formed wood plastic composite product. Proc. CIRP.

[48] Ayrilmis N., Benthien J.T., Thoemen H. (2012). Effects of formulation variables on surface properties of wood plastic composites. Compos., Part B.

[49] Toghyani A.E., Matthews S., Eskelinen H., Kärki T., Varis J. (2016). Feasibility assessment of a wood-plastic composite post-production process: formability. Bioresources.

